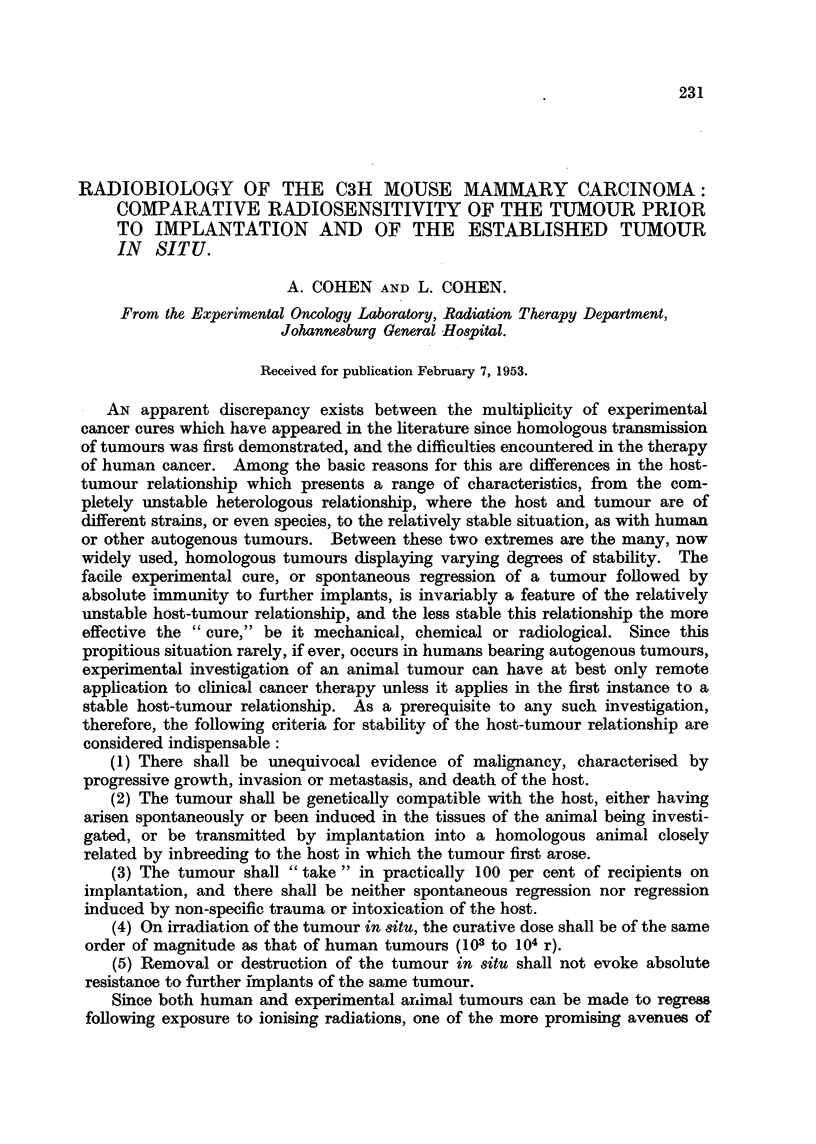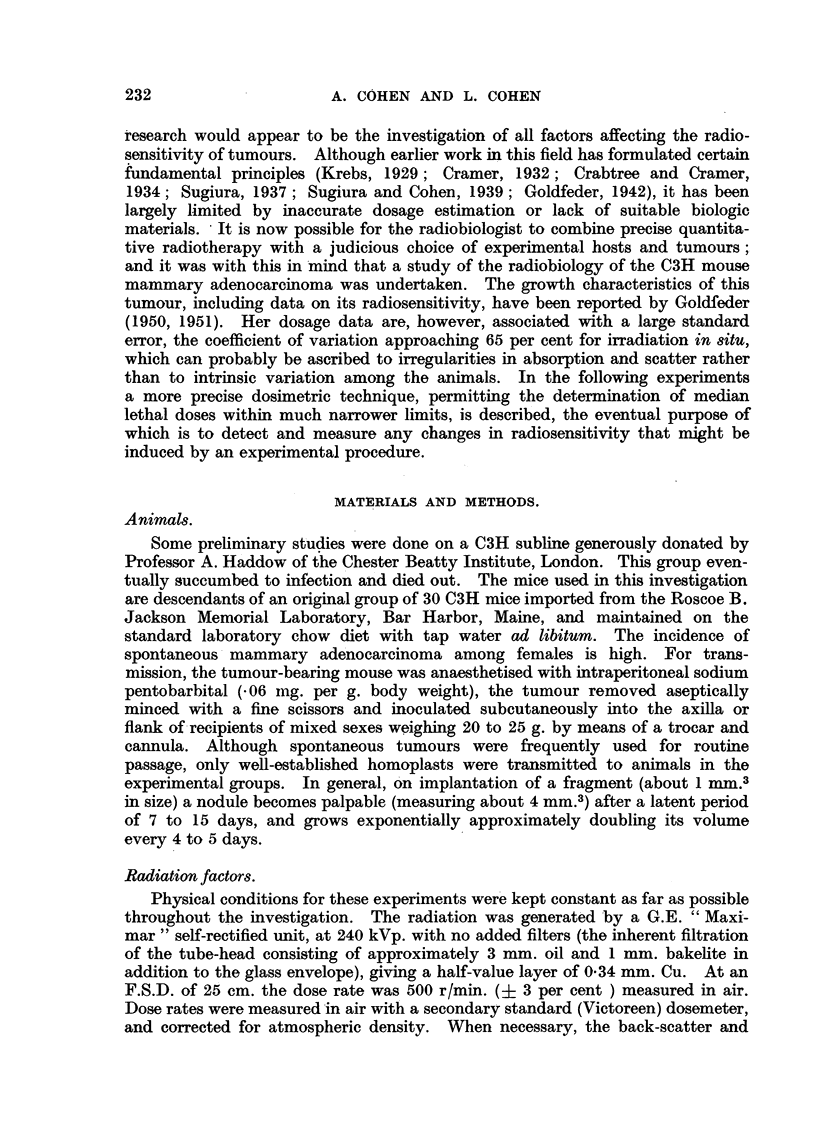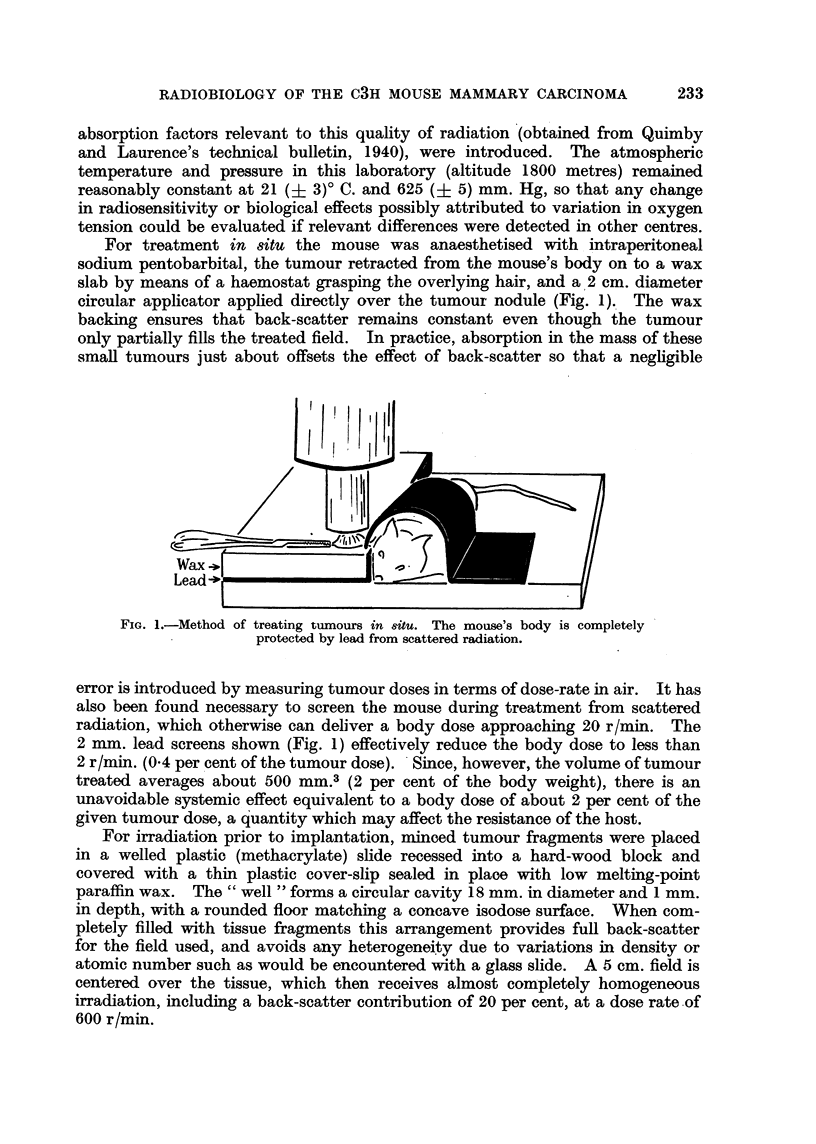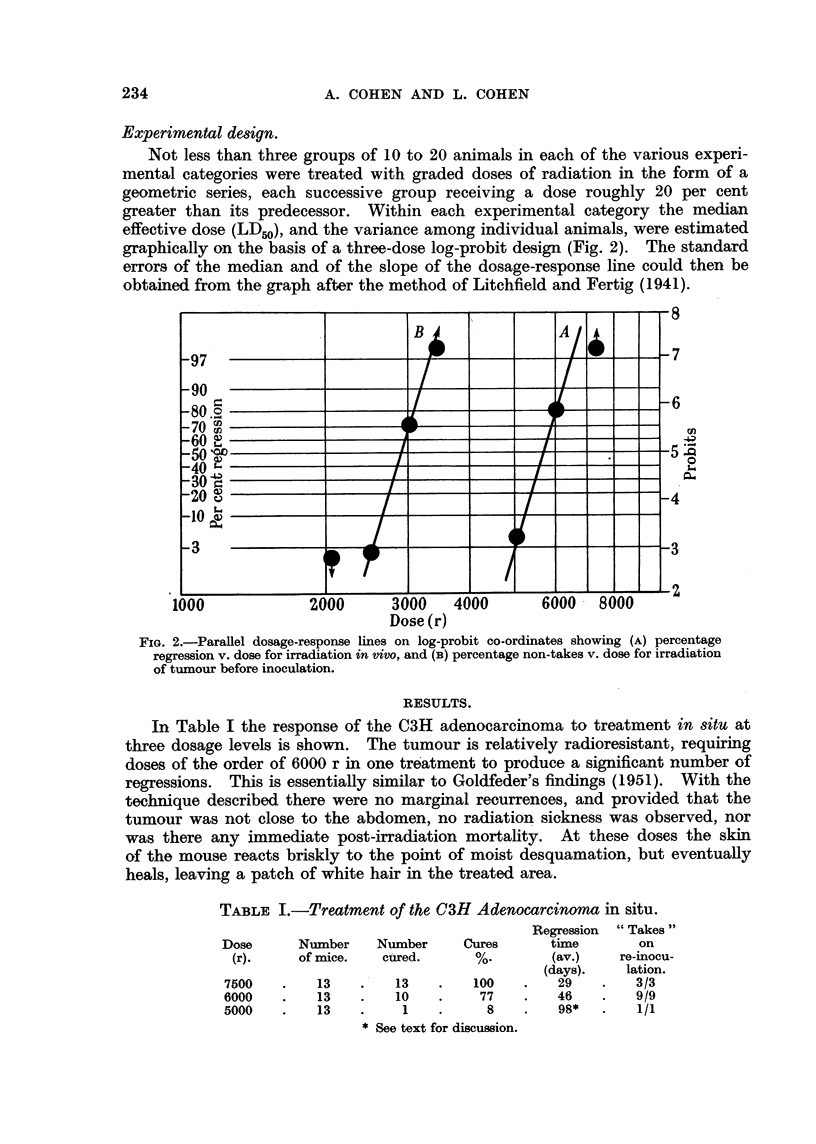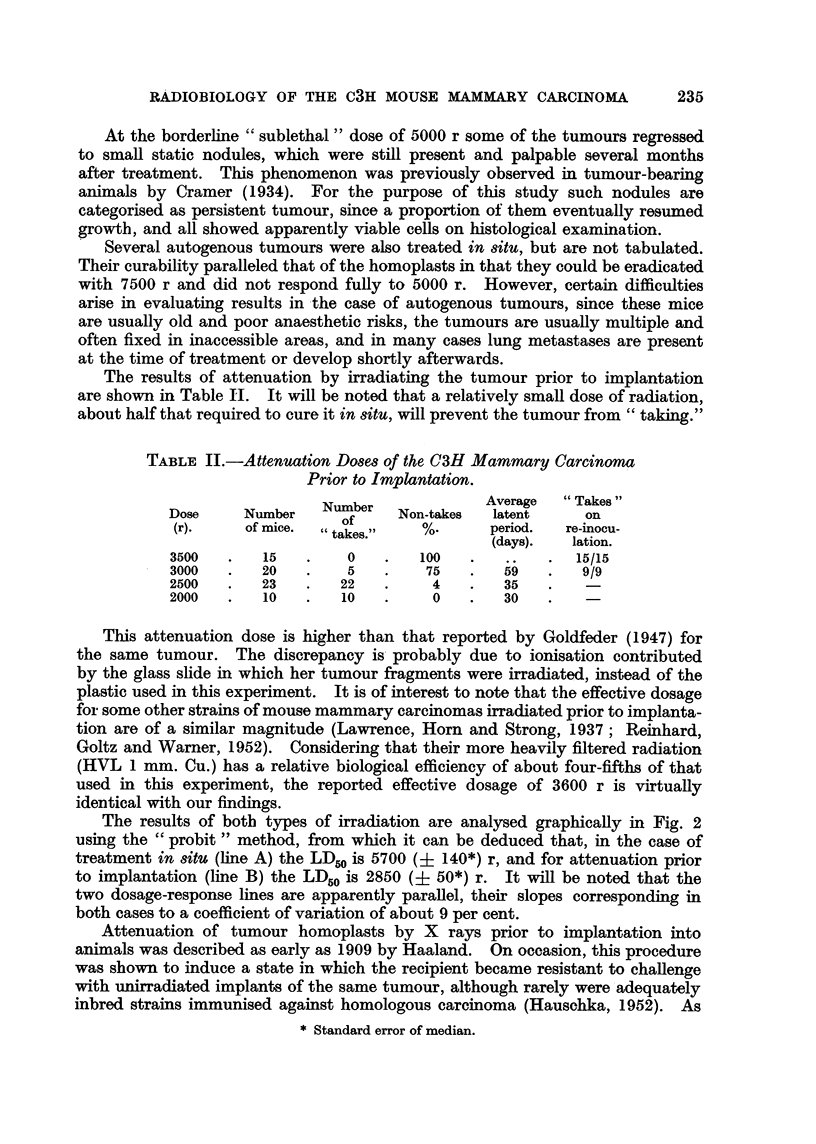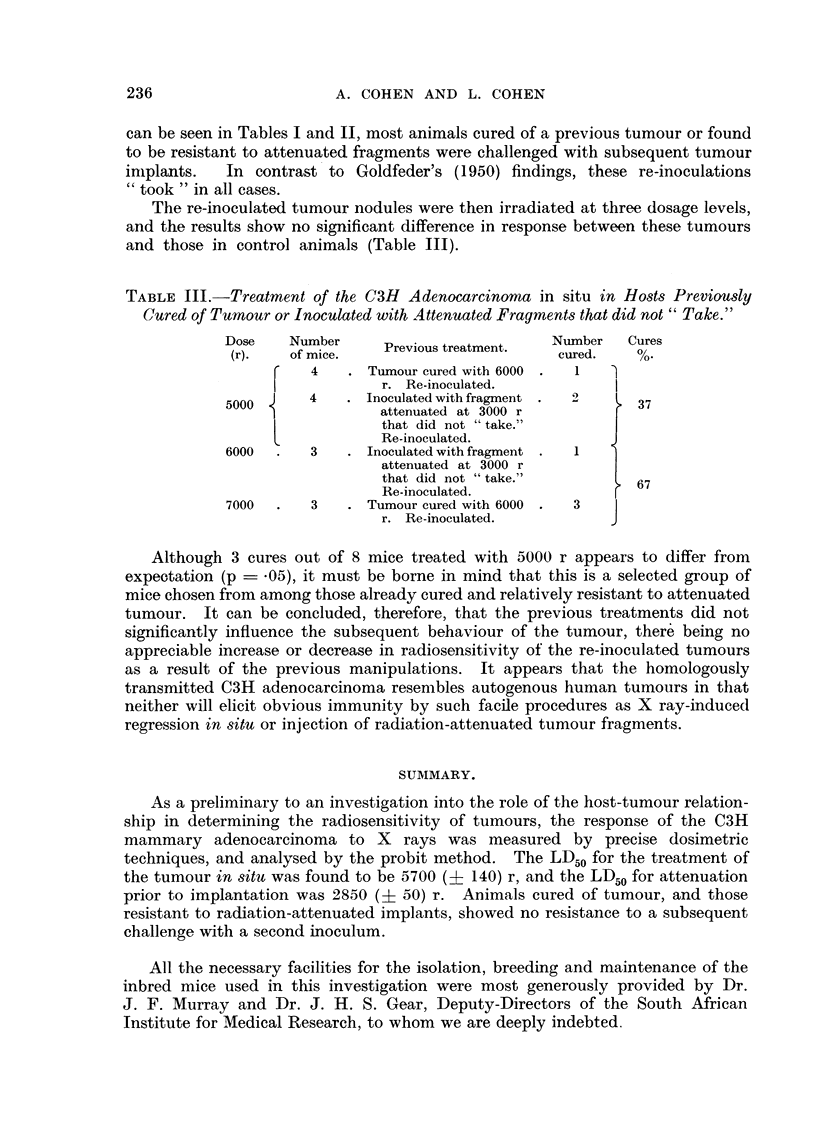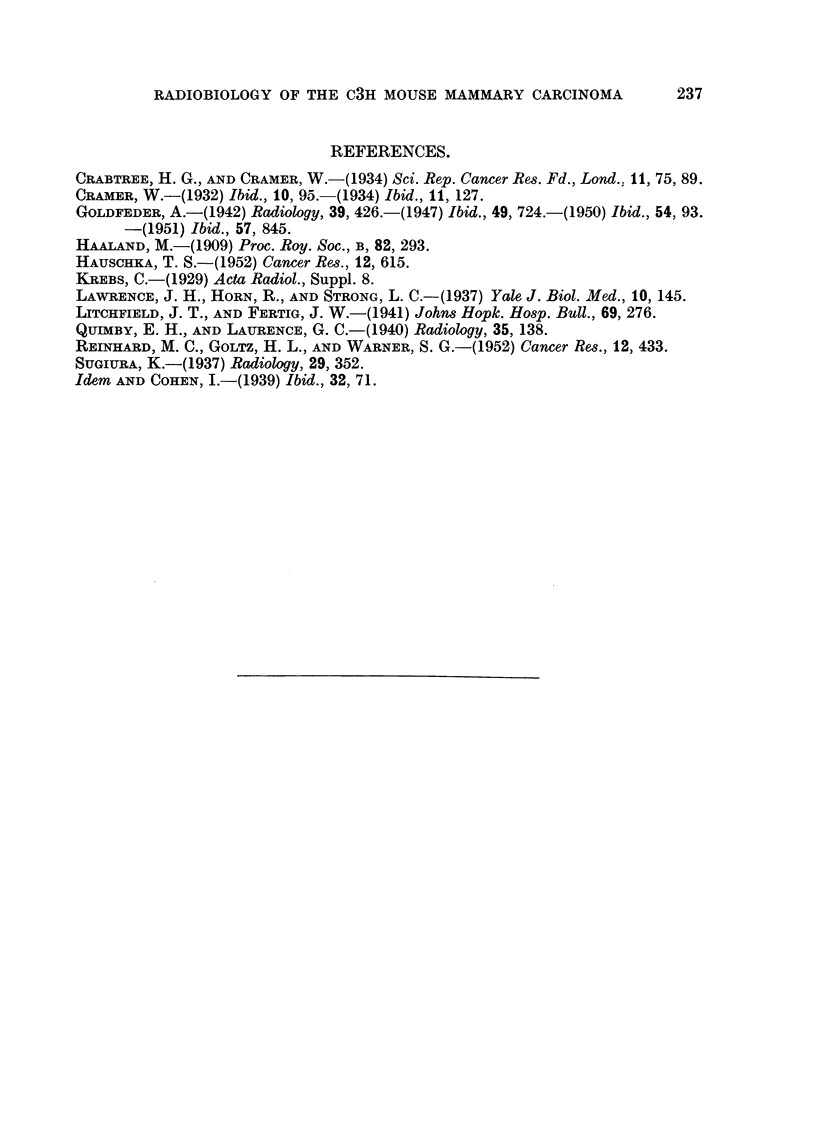# Radiobiology of the C3H Mouse Mammary Carcinoma: Comparative Radiosensitivity of the Tumour Prior to Implantation and of the Established Tumour In Situ

**DOI:** 10.1038/bjc.1953.21

**Published:** 1953-06

**Authors:** A. Cohen, L. Cohen


### RADIOBIOLOGY OF THE C3H MOUSE MAMMARY CARCINOMA: COMPARATIVE RADIOSENSITIVITY OF THE TUMOUR PRIOR TO IMPLANTATION AND OF THE ESTABLISHED TUMOUR *IN SITU*.

A. COHEN AND L. COHEN.

*From the Experimental Oncology Laboratory, Radiation Therapy Department, Johannesburg General Hospital.*

Received for publication February 7, 1953.

An apparent discrepancy exists between the multiplicity of experimental cancer cures which have appeared in the literature since homologous transmission of tumours was first demonstrated, and the difficulties encountered in the therapy of human cancer. Among the basic reasons for this are differences in the host-tumour relationship which presents a range of characteristics, from the completely unstable heterologous relationship, where the host and tumour are of different strains, or even species, to the relatively stable situation, as with human or other autogenous tumours. Between these two extremes are the many, now widely used, homologous tumours displaying varying degrees of stability. The facile experimental cure, or spontaneous regression of a tumour followed by absolute immunity to further implants, is invariably a feature of the relatively unstable host-tumour relationship, and the less stable this relationship the more effective the "cure," be it mechanical, chemical or radiological. Since this propitious situation rarely, if ever, occurs in humans bearing autogenous tumours, experimental investigation of an animal tumour can have at best only remote application to clinical cancer therapy unless it applies in the first instance to a stable host-tumour relationship. As a prerequisite to any such investigation, therefore, the following criteria for stability of the host-tumour relationship are considered indispensable:

(1) There shall be unequivocal evidence of malignancy, characterised by progressive growth, invasion or metastasis, and death of the host.

(2) The tumour shall be genetically compatible with the host, either having arisen spontaneously or been induced in the tissues of the animal being investigated, or be transmitted by implantation into a homologous animal closely related by inbreeding to the host in which the tumour first arose.

(3) The tumour shall "take" in practically 100 per cent of recipients on implantation, and there shall be neither spontaneous regression nor regression induced by non-specific trauma or intoxication of the host.

(4) On irradiation of the tumour *in situ*, the curative dose shall be of the same order of magnitude as that of human tumours ($10^3$ to $10^4$ r).

(5) Removal or destruction of the tumour *in situ* shall not evoke absolute resistance to further implants of the same tumour.

Since both human and experimental animal tumours can be made to regress following exposure to ionising radiations, one of the more promising avenues of research would appear to be the investigation of all factors affecting the radiosensitivity of tumours. Although earlier work in this field has formulated certain fundamental principles (Krebs, 1929; Cramer, 1932; Crabtree and Cramer, 1934; Sugiura, 1937; Sugiura and Cohen, 1939; Goldfeder, 1942), it has been largely limited by inaccurate dosage estimation or lack of suitable biologic materials. It is now possible for the radiobiologist to combine precise quantitative radiotherapy with a judicious choice of experimental hosts and tumours; and it was with this in mind that a study of the radiobiology of the C3H mouse mammary adenocarcinoma was undertaken. The growth characteristics of this tumour, including data on its radiosensitivity, have been reported by Goldfeder (1950, 1951). Her dosage data are, however, associated with a large standard error, the coefficient of variation approaching 65 per cent for irradiation *in situ*, which can probably be ascribed to irregularities in absorption and scatter rather than to intrinsic variation among the animals. In the following experiments a more precise dosimetric technique, permitting the determination of median lethal doses within much narrower limits, is described, the eventual purpose of which is to detect and measure any changes in radiosensitivity that might be induced by an experimental procedure.

### MATERIALS AND METHODS.

### *Animals.*

Some preliminary studies were done on a C3H subline generously donated by Professor A. Haddow of the Chester Beatty Institute, London. This group eventually succumbed to infection and died out. The mice used in this investigation are descendants of an original group of 30 C3H mice imported from the Roscoe B. Jackson Memorial Laboratory, Bar Harbor, Maine, and maintained on the standard laboratory chow diet with tap water *ad libitum*. The incidence of spontaneous mammary adenocarcinoma among females is high. For transmission, the tumour-bearing mouse was anaesthetised with intraperitoneal sodium pentobarbital (·06 mg. per g. body weight), the tumour removed aseptically minced with a fine scissors and inoculated subcutaneously into the axilla or flank of recipients of mixed sexes weighing 20 to 25 g. by means of a trocar and cannula. Although spontaneous tumours were frequently used for routine passage, only well-established homoplasts were transmitted to animals in the experimental groups. In general, on implantation of a fragment (about 1 $mm.^3$ in size) a nodule becomes palpable (measuring about 4 $mm.^3$) after a latent period of 7 to 15 days, and grows exponentially approximately doubling its volume every 4 to 5 days.

### *Radiation factors.*

Physical conditions for these experiments were kept constant as far as possible throughout the investigation. The radiation was generated by a G.E. "Maximar" self-rectified unit, at 240 kVp. with no added filters (the inherent filtration of the tube-head consisting of approximately 3 mm. oil and 1 mm. bakelite in addition to the glass envelope), giving a half-value layer of 0·34 mm. Cu. At an F.S.D. of 25 cm. the dose rate was 500 r/min. ($\pm$ 3 per cent ) measured in air. Dose rates were measured in air with a secondary standard (Victoreen) dosemeter, and corrected for atmospheric density. When necessary, the back-scatter and absorption factors relevant to this quality of radiation (obtained from Quimby and Laurence's technical bulletin, 1940), were introduced. The atmospheric temperature and pressure in this laboratory (altitude 1800 metres) remained reasonably constant at 21 ($\pm$ 3)° C. and 625 ($\pm$ 5) mm. Hg, so that any change in radiosensitivity or biological effects possibly attributed to variation in oxygen tension could be evaluated if relevant differences were detected in other centres.

For treatment *in situ* the mouse was anaesthetised with intraperitoneal sodium pentobarbital, the tumour retracted from the mouse's body on to a wax slab by means of a haemostat grasping the overlying hair, and a 2 cm. diameter circular applicator applied directly over the tumour nodule (Fig. 1). The wax backing ensures that back-scatter remains constant even though the tumour only partially fills the treated field. In practice, absorption in the mass of these small tumours just about offsets the effect of back-scatter so that a negligible

FIG. 1.—Method of treating tumours *in situ*. The mouse's body is completely protected by lead from scattered radiation.

error is introduced by measuring tumour doses in terms of dose-rate in air. It has also been found necessary to screen the mouse during treatment from scattered radiation, which otherwise can deliver a body dose approaching 20 r/min. The 2 mm. lead screens shown (Fig. 1) effectively reduce the body dose to less than 2 r/min. (0·4 per cent of the tumour dose). Since, however, the volume of tumour treated averages about 500 mm.$^3$ (2 per cent of the body weight), there is an unavoidable systemic effect equivalent to a body dose of about 2 per cent of the given tumour dose, a quantity which may affect the resistance of the host.

For irradiation prior to implantation, minced tumour fragments were placed in a welled plastic (methacrylate) slide recessed into a hard-wood block and covered with a thin plastic cover-slip sealed in place with low melting-point paraffin wax. The " well " forms a circular cavity 18 mm. in diameter and 1 mm. in depth, with a rounded floor matching a concave isodose surface. When completely filled with tissue fragments this arrangement provides full back-scatter for the field used, and avoids any heterogeneity due to variations in density or atomic number such as would be encountered with a glass slide. A 5 cm. field is centered over the tissue, which then receives almost completely homogeneous irradiation, including a back-scatter contribution of 20 per cent, at a dose rate of 600 r/min.

*Experimental design.*

Not less than three groups of 10 to 20 animals in each of the various experimental categories were treated with graded doses of radiation in the form of a geometric series, each successive group receiving a dose roughly 20 per cent greater than its predecessor. Within each experimental category the median effective dose ($LD_{50}$), and the variance among individual animals, were estimated graphically on the basis of a three-dose log-probit design (Fig. 2). The standard errors of the median and of the slope of the dosage-response line could then be obtained from the graph after the method of Litchfield and Fertig (1941).

FIG. 2.—Parallel dosage-response lines on log-probit co-ordinates showing (A) percentage regression v. dose for irradiation *in vivo*, and (B) percentage non-takes v. dose for irradiation of tumour before inoculation.

### RESULTS.

In Table I the response of the C3H adenocarcinoma to treatment *in situ* at three dosage levels is shown. The tumour is relatively radioresistant, requiring doses of the order of 6000 r in one treatment to produce a significant number of regressions. This is essentially similar to Goldfeder's findings (1951). With the technique described there were no marginal recurrences, and provided that the tumour was not close to the abdomen, no radiation sickness was observed, nor was there any immediate post-irradiation mortality. At these doses the skin of the mouse reacts briskly to the point of moist desquamation, but eventually heals, leaving a patch of white hair in the treated area.

TABLE I.—*Treatment of the C3H Adenocarcinoma* in situ.

| Dose (r). | Number of mice. | Number cured. | Cures %. | Regression time (av.) (days). | " Takes " on re-inoculation. |
|---|---|---|---|---|---|
| 7500 | 13 | 13 | 100 | 29 | 3/3 |
| 6000 | 13 | 10 | 77 | 46 | 9/9 |
| 5000 | 13 | 1 | 8 | 98* | 1/1 |

* See text for discussion.

At the borderline " sublethal " dose of 5000 r some of the tumours regressed to small static nodules, which were still present and palpable several months after treatment. This phenomenon was previously observed in tumour-bearing animals by Cramer (1934). For the purpose of this study such nodules are categorised as persistent tumour, since a proportion of them eventually resumed growth, and all showed apparently viable cells on histological examination.

Several autogenous tumours were also treated *in situ*, but are not tabulated. Their curability paralleled that of the homoplasts in that they could be eradicated with 7500 r and did not respond fully to 5000 r. However, certain difficulties arise in evaluating results in the case of autogenous tumours, since these mice are usually old and poor anaesthetic risks, the tumours are usually multiple and often fixed in inaccessible areas, and in many cases lung metastases are present at the time of treatment or develop shortly afterwards.

The results of attenuation by irradiating the tumour prior to implantation are shown in Table II. It will be noted that a relatively small dose of radiation, about half that required to cure it *in situ*, will prevent the tumour from " taking."

TABLE II.—*Attenuation Doses of the C3H Mammary Carcinoma Prior to Implantation.*

| Dose (r). | Number of mice. | Number of " takes." | Non-takes %. | Average latent period. (days). | " Takes " on re-inoculation. |
|---|---|---|---|---|---|
| 3500 | 15 | 0 | 100 | .. | 15/15 |
| 3000 | 20 | 5 | 75 | 59 | 9/9 |
| 2500 | 23 | 22 | 4 | 35 | — |
| 2000 | 10 | 10 | 0 | 30 | — |

This attenuation dose is higher than that reported by Goldfeder (1947) for the same tumour. The discrepancy is probably due to ionisation contributed by the glass slide in which her tumour fragments were irradiated, instead of the plastic used in this experiment. It is of interest to note that the effective dosage for some other strains of mouse mammary carcinomas irradiated prior to implantation are of a similar magnitude (Lawrence, Horn and Strong, 1937 ; Reinhard, Goltz and Warner, 1952). Considering that their more heavily filtered radiation (HVL 1 mm. Cu.) has a relative biological efficiency of about four-fifths of that used in this experiment, the reported effective dosage of 3600 r is virtually identical with our findings.

The results of both types of irradiation are analysed graphically in Fig. 2 using the " probit " method, from which it can be deduced that, in the case of treatment *in situ* (line A) the $LD_{50}$ is 5700 ($\pm$ 140*) r, and for attenuation prior to implantation (line B) the $LD_{50}$ is 2850 ($\pm$ 50*) r. It will be noted that the two dosage-response lines are apparently parallel, their slopes corresponding in both cases to a coefficient of variation of about 9 per cent.

Attenuation of tumour homoplasts by X rays prior to implantation into animals was described as early as 1909 by Haaland. On occasion, this procedure was shown to induce a state in which the recipient became resistant to challenge with unirradiated implants of the same tumour, although rarely were adequately inbred strains immunised against homologous carcinoma (Hauschka, 1952). As

* Standard error of median.

can be seen in Tables I and II, most animals cured of a previous tumour or found to be resistant to attenuated fragments were challenged with subsequent tumour implants. In contrast to Goldfeder's (1950) findings, these re-inoculations "took" in all cases.

The re-inoculated tumour nodules were then irradiated at three dosage levels, and the results show no significant difference in response between these tumours and those in control animals (Table III).

TABLE III.—*Treatment of the C3H Adenocarcinoma* in situ *in Hosts Previously Cured of Tumour or Inoculated with Attenuated Fragments that did not "Take."*

| Dose (r). | Number of mice. | Previous treatment. | Number cured. | Cures %. |
|---|---|---|---|---|
| 5000 | 4 | Tumour cured with 6000 r. Re-inoculated. | 1 | 37 |
| | 4 | Inoculated with fragment attenuated at 3000 r that did not "take." Re-inoculated. | 2 | |
| 6000 | 3 | Inoculated with fragment attenuated at 3000 r that did not "take." Re-inoculated. | 1 | 67 |
| 7000 | 3 | Tumour cured with 6000 r. Re-inoculated. | 3 | |

Although 3 cures out of 8 mice treated with 5000 r appears to differ from expectation ($p = \cdot 05$), it must be borne in mind that this is a selected group of mice chosen from among those already cured and relatively resistant to attenuated tumour. It can be concluded, therefore, that the previous treatments did not significantly influence the subsequent behaviour of the tumour, there being no appreciable increase or decrease in radiosensitivity of the re-inoculated tumours as a result of the previous manipulations. It appears that the homologously transmitted C3H adenocarcinoma resembles autogenous human tumours in that neither will elicit obvious immunity by such facile procedures as X ray-induced regression *in situ* or injection of radiation-attenuated tumour fragments.

### SUMMARY.

As a preliminary to an investigation into the role of the host-tumour relationship in determining the radiosensitivity of tumours, the response of the C3H mammary adenocarcinoma to X rays was measured by precise dosimetric techniques, and analysed by the probit method. The $LD_{50}$ for the treatment of the tumour *in situ* was found to be 5700 ($\pm$ 140) r, and the $LD_{50}$ for attenuation prior to implantation was 2850 ($\pm$ 50) r. Animals cured of tumour, and those resistant to radiation-attenuated implants, showed no resistance to a subsequent challenge with a second inoculum.

All the necessary facilities for the isolation, breeding and maintenance of the inbred mice used in this investigation were most generously provided by Dr. J. F. Murray and Dr. J. H. S. Gear, Deputy-Directors of the South African Institute for Medical Research, to whom we are deeply indebted.

### REFERENCES.

CRABTREE, H. G., AND CRAMER, W.—(1934) *Sci. Rep. Cancer Res. Fd., Lond.*, **11**, 75, 89.
CRAMER, W.—(1932) *Ibid.*, **10**, 95.—(1934) *Ibid.*, **11**, 127.
GOLDFEDER, A.—(1942) *Radiology*, **39**, 426.—(1947) *Ibid.*, **49**, 724.—(1950) *Ibid.*, **54**, 93. —(1951) *Ibid.*, **57**, 845.
HAALAND, M.—(1909) *Proc. Roy. Soc.*, B, **82**, 293.
HAUSCHKA, T. S.—(1952) *Cancer Res.*, **12**, 615.
KREBS, C.—(1929) *Acta Radiol.*, Suppl. 8.
LAWRENCE, J. H., HORN, R., AND STRONG, L. C.—(1937) *Yale J. Biol. Med.*, **10**, 145.
LITCHFIELD, J. T., AND FERTIG, J. W.—(1941) *Johns Hopk. Hosp. Bull.*, **69**, 276.
QUIMBY, E. H., AND LAURENCE, G. C.—(1940) *Radiology*, **35**, 138.
REINHARD, M. C., GOLTZ, H. L., AND WARNER, S. G.—(1952) *Cancer Res.*, **12**, 433.
SUGIURA, K.—(1937) *Radiology*, **29**, 352.
*Idem* AND COHEN, I.—(1939) *Ibid.*, **32**, 71.